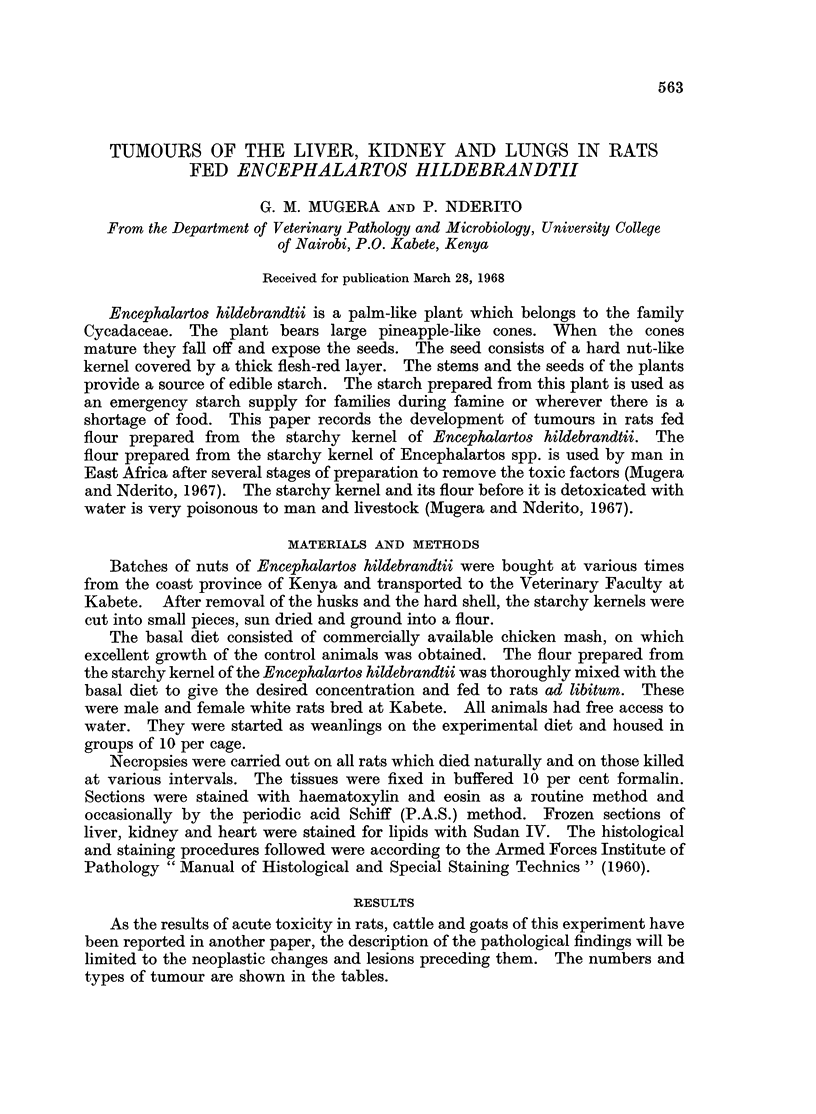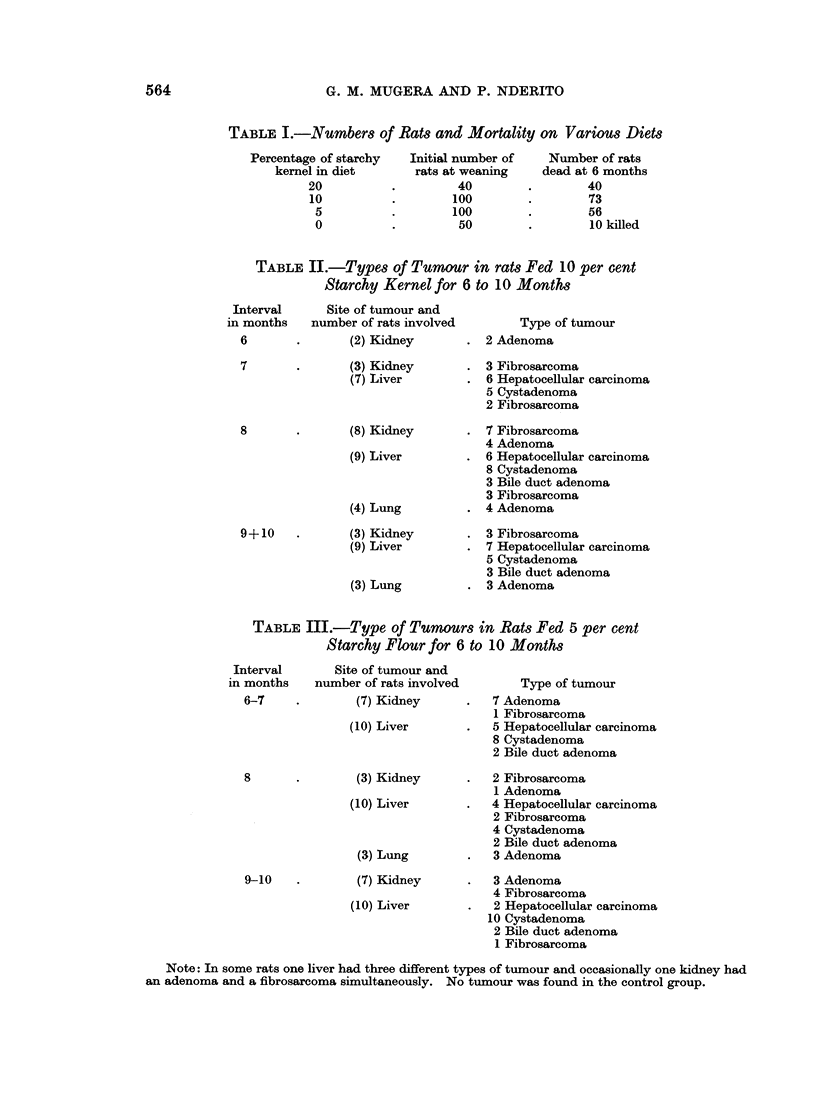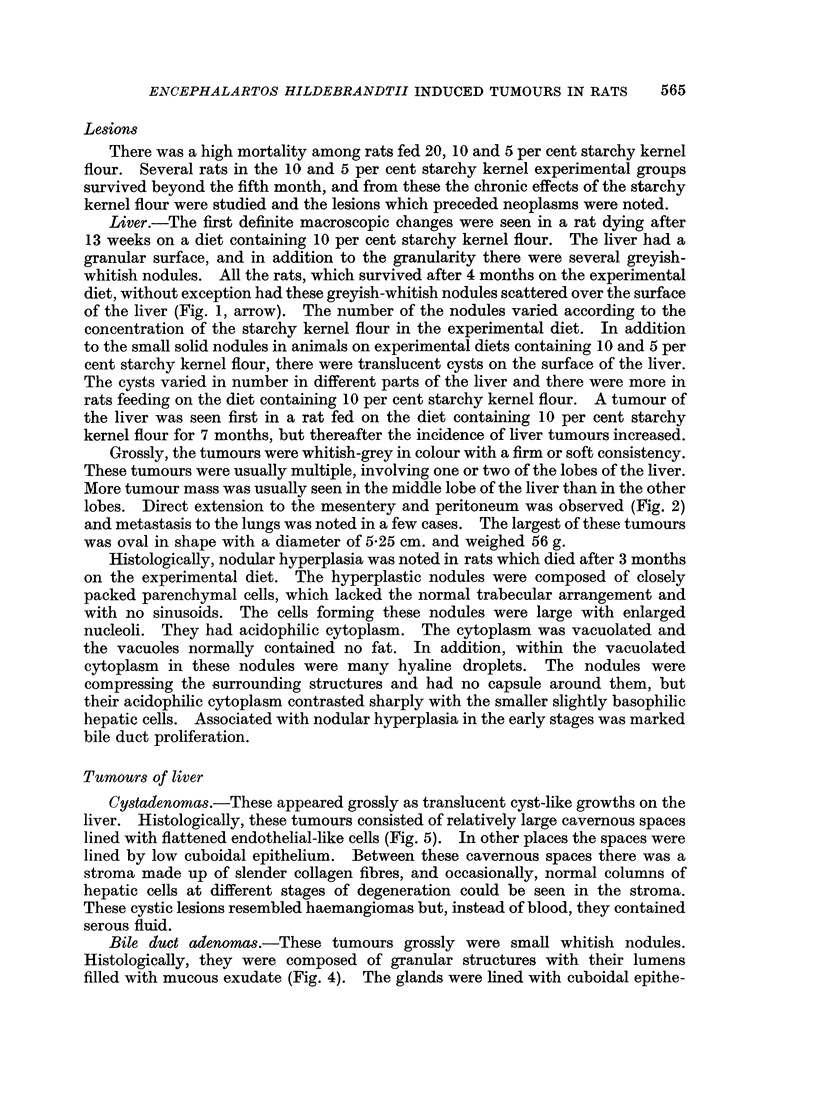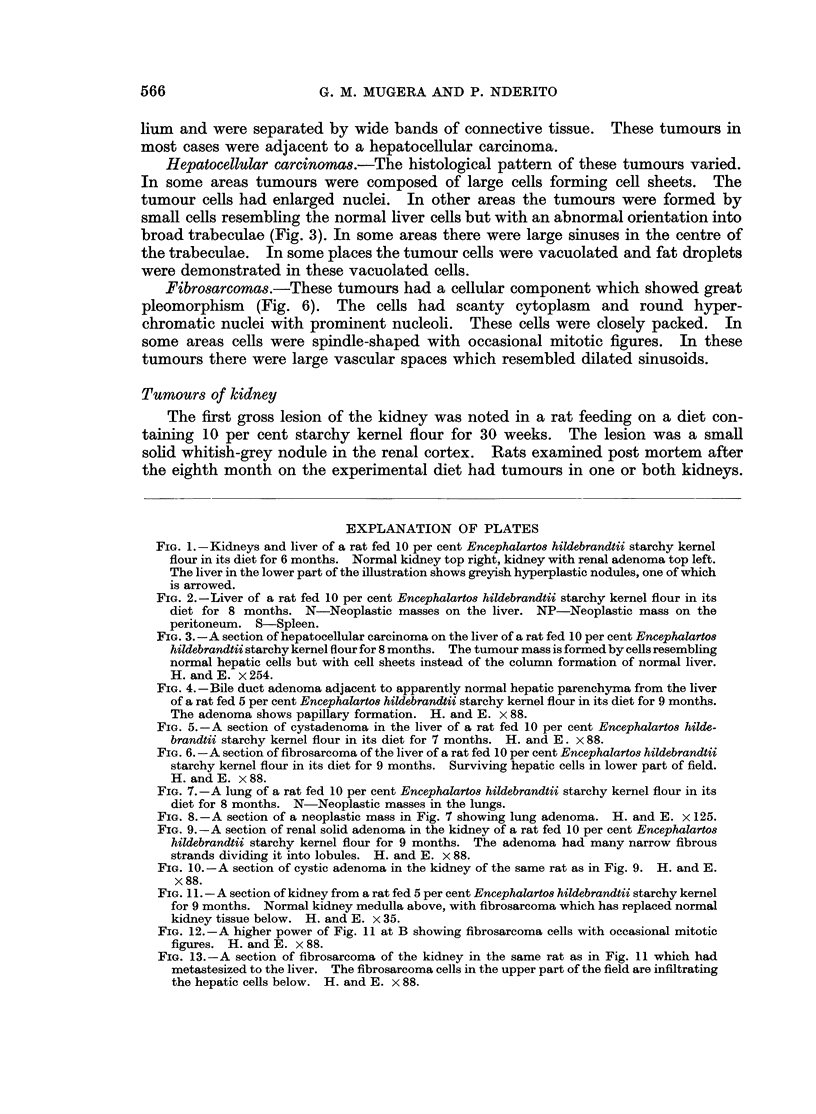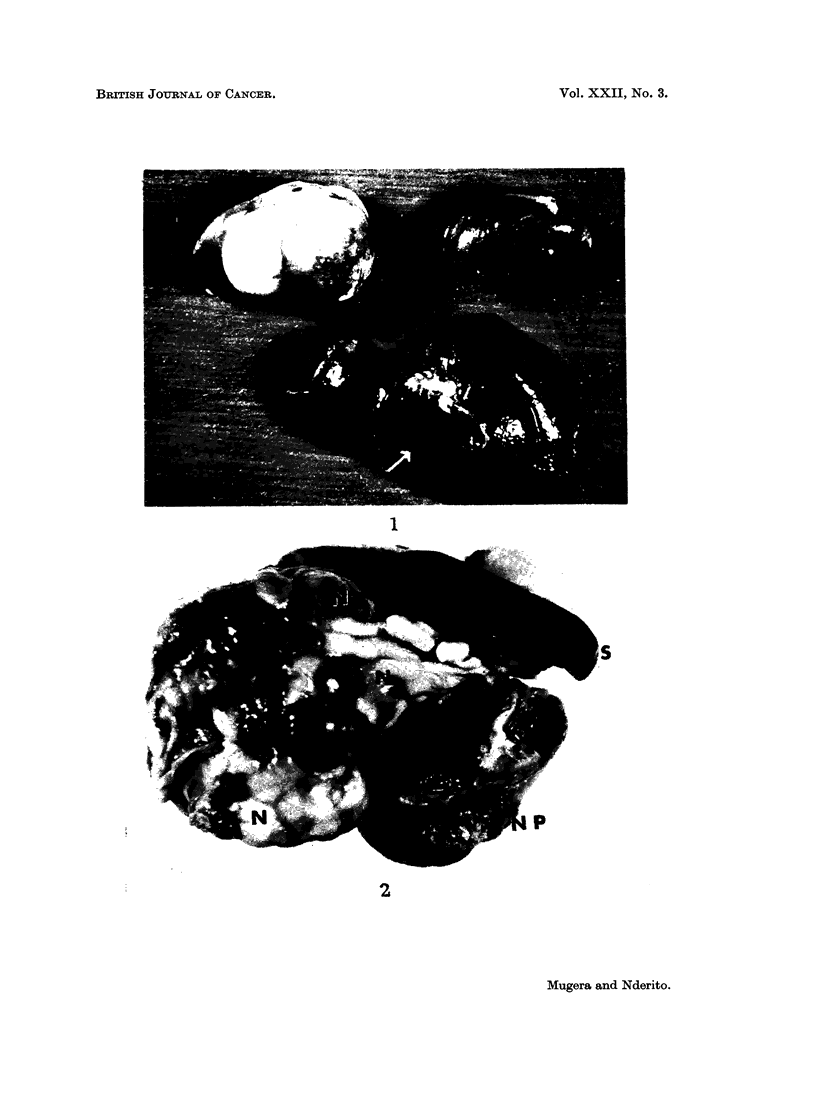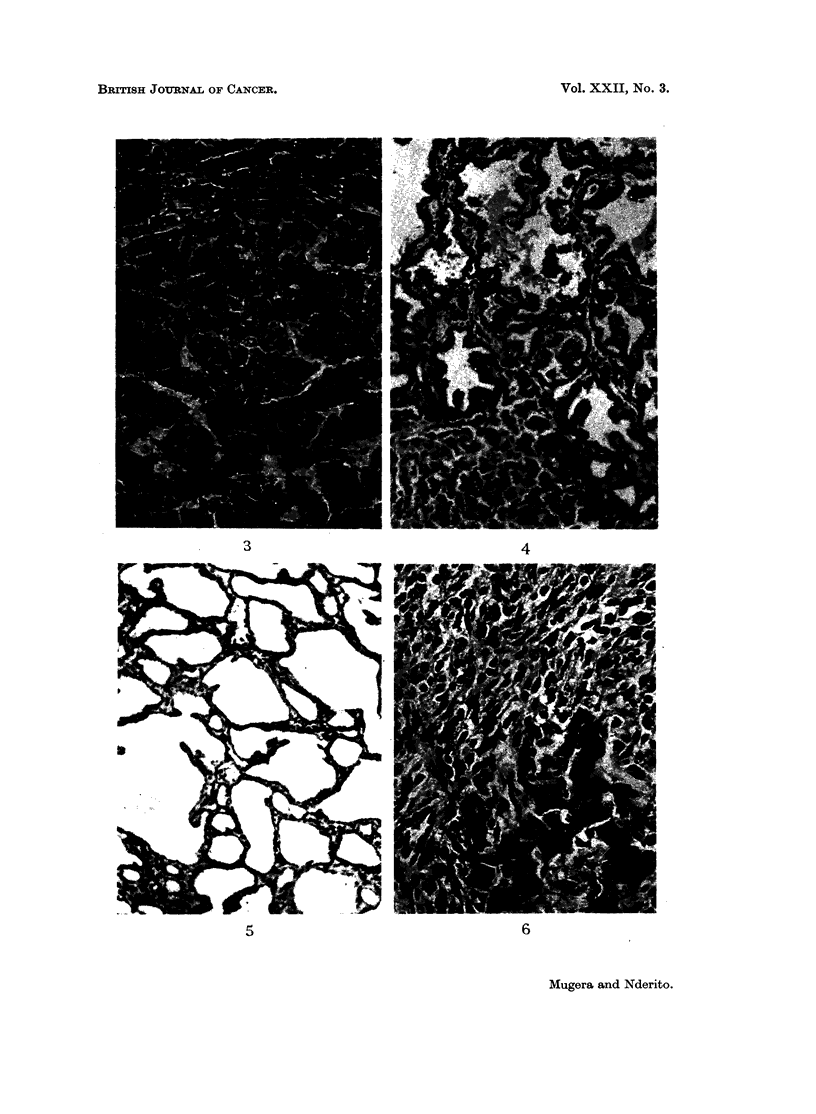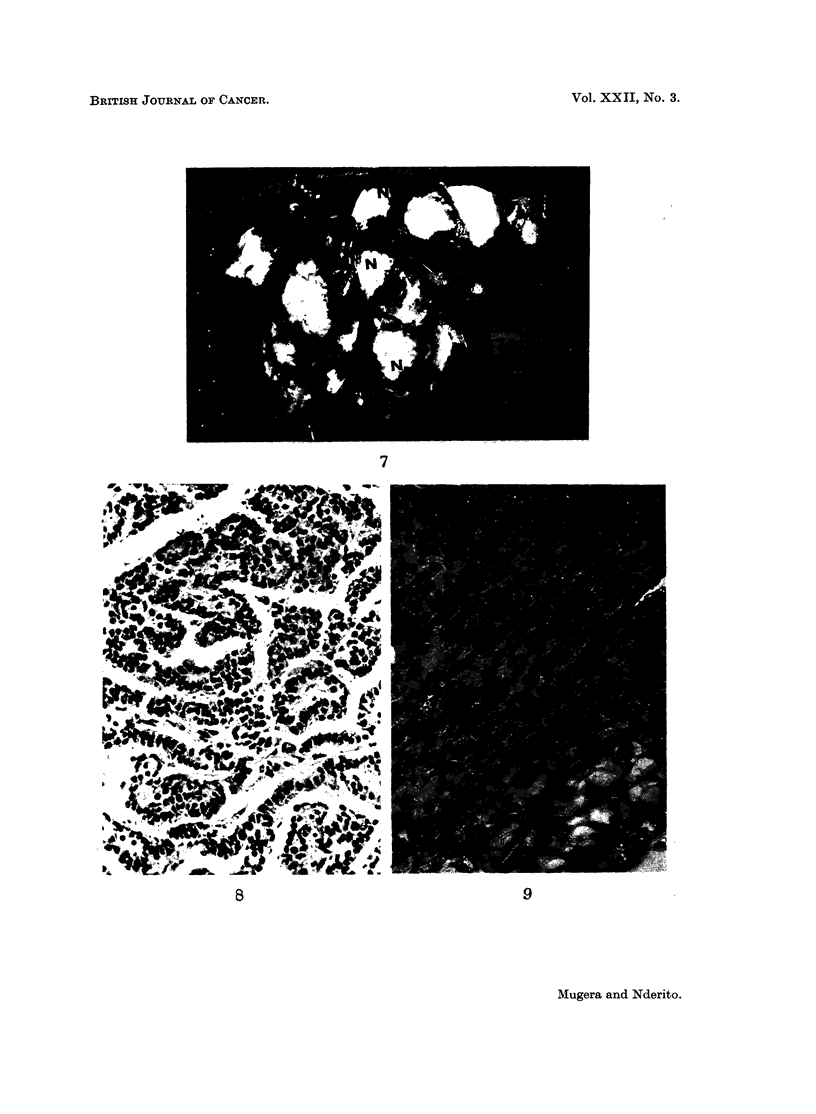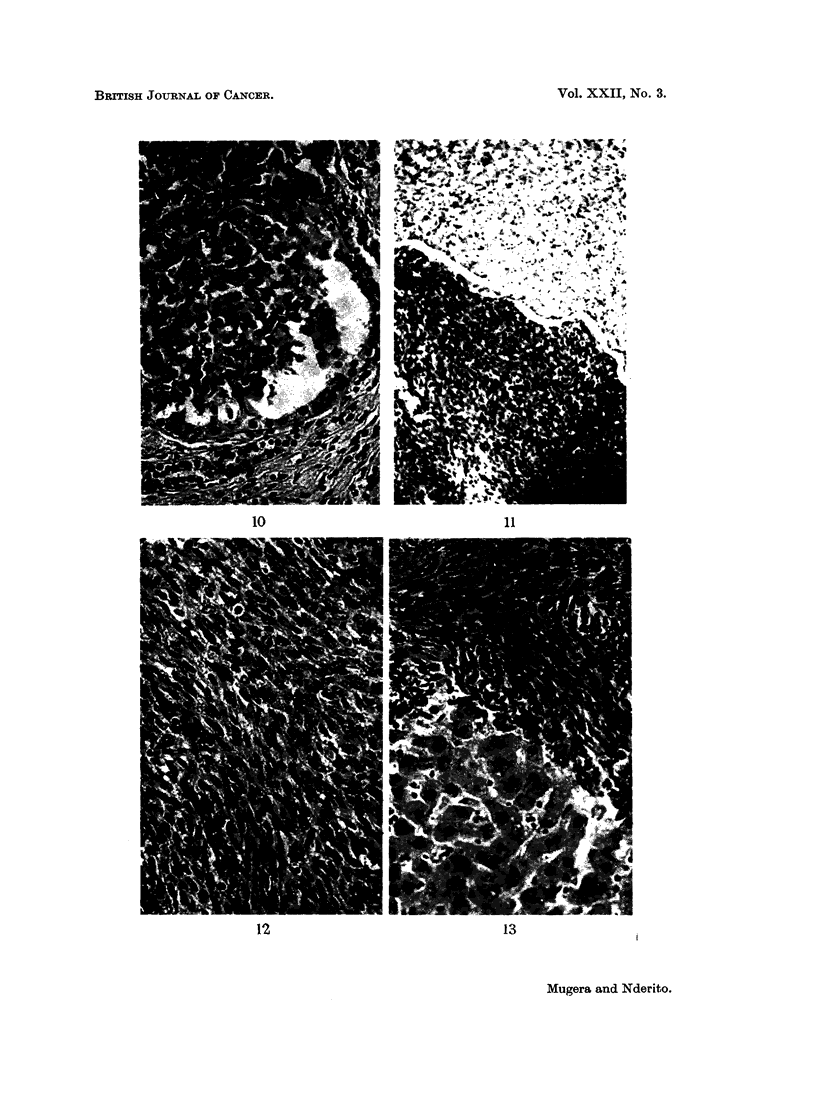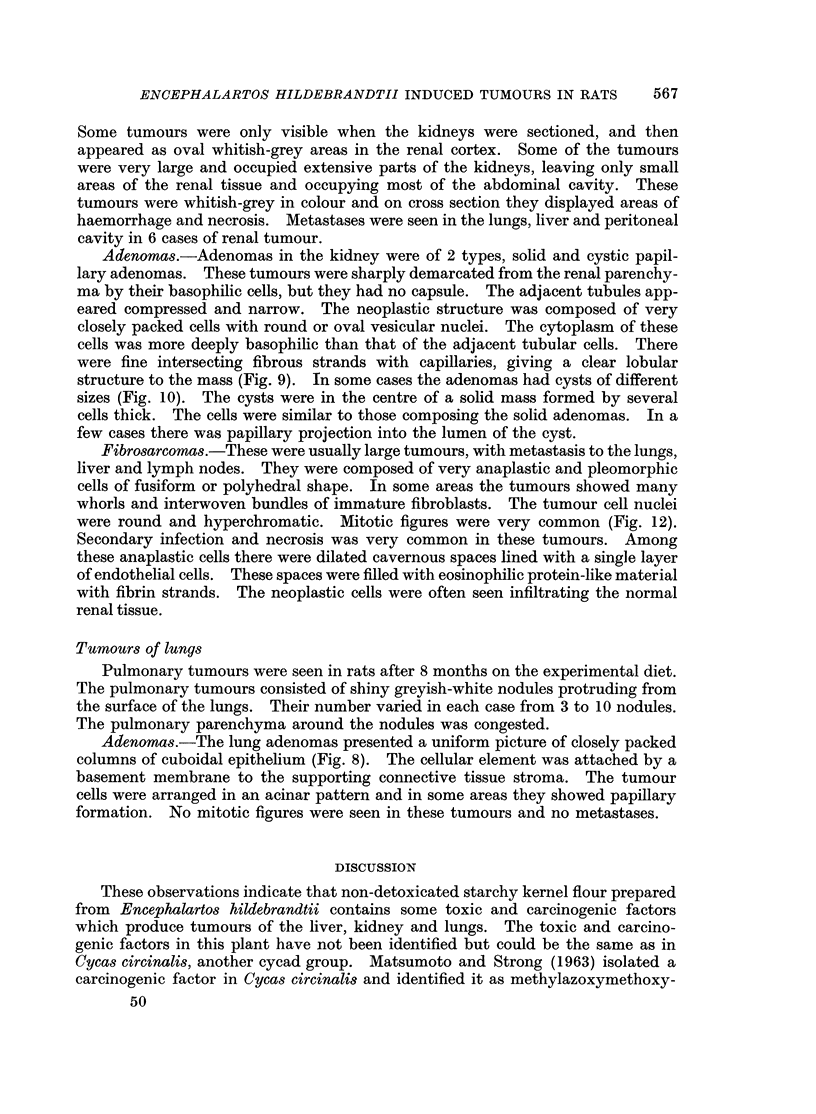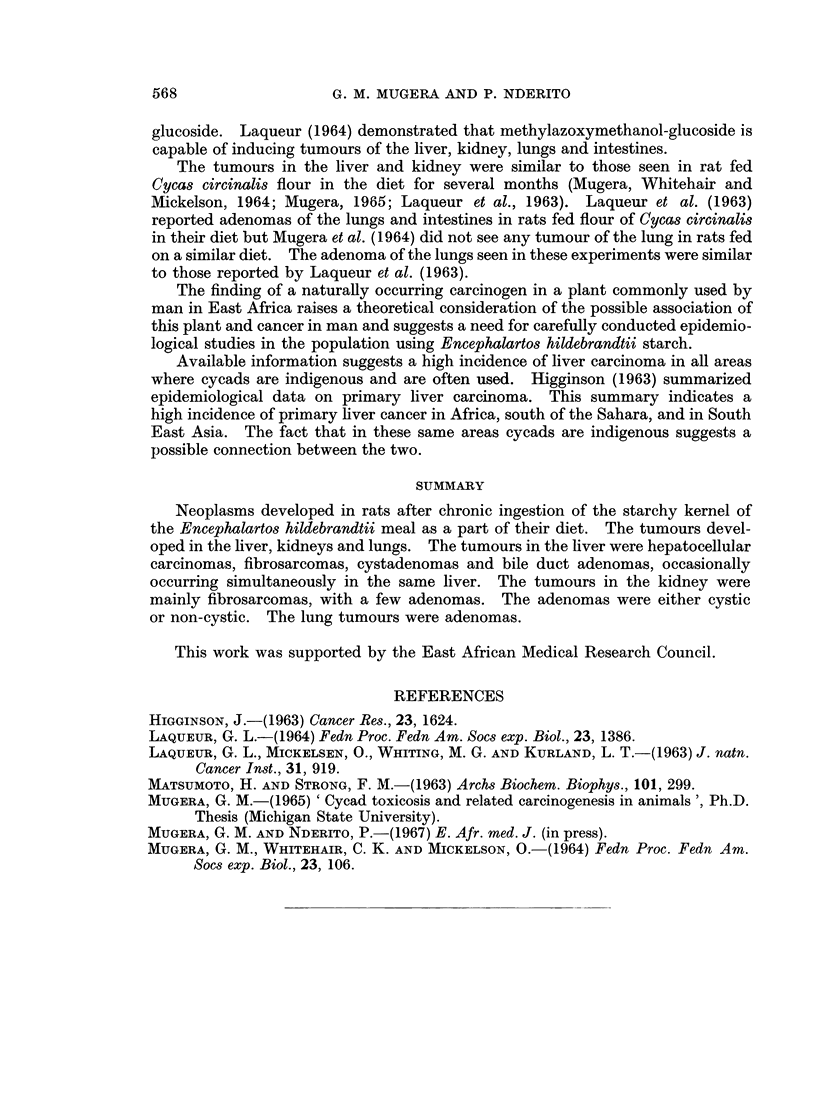# Tumours of the liver, kidney and lungs in rats fed Encephalartos hildebrandtii.

**DOI:** 10.1038/bjc.1968.66

**Published:** 1968-09

**Authors:** G. M. Mugera, P. Nderito

## Abstract

**Images:**


### TUMOURS OF THE LIVER, KIDNEY AND LUNGS IN RATS FED *ENCEPHALARTOS HILDEBRANDTII*

G. M. MUGERA AND P. NDERITO

*From the Department of Veterinary Pathology and Microbiology, University College of Nairobi, P.O. Kabete, Kenya*

Received for publication March 28, 1968

*Encephalartos hildebrandtii* is a palm-like plant which belongs to the family Cycadaceae. The plant bears large pineapple-like cones. When the cones mature they fall off and expose the seeds. The seed consists of a hard nut-like kernel covered by a thick flesh-red layer. The stems and the seeds of the plants provide a source of edible starch. The starch prepared from this plant is used as an emergency starch supply for families during famine or wherever there is a shortage of food. This paper records the development of tumours in rats fed flour prepared from the starchy kernel of *Encephalartos hildebrandtii.* The flour prepared from the starchy kernel of Encephalartos spp. is used by man in East Africa after several stages of preparation to remove the toxic factors (Mugera and Nderito, 1967). The starchy kernel and its flour before it is detoxicated with water is very poisonous to man and livestock (Mugera and Nderito, 1967).

### MATERIALS AND METHODS

Batches of nuts of *Encephalartos hildebrandtii* were bought at various times from the coast province of Kenya and transported to the Veterinary Faculty at Kabete. After removal of the husks and the hard shell, the starchy kernels were cut into small pieces, sun dried and ground into a flour.

The basal diet consisted of commercially available chicken mash, on which excellent growth of the control animals was obtained. The flour prepared from the starchy kernel of the *Encephalartos hildebrandtii* was thoroughly mixed with the basal diet to give the desired concentration and fed to rats *ad libitum.* These were male and female white rats bred at Kabete. All animals had free access to water. They were started as weanlings on the experimental diet and housed in groups of 10 per cage.

Necropsies were carried out on all rats which died naturally and on those killed at various intervals. The tissues were fixed in buffered 10 per cent formalin. Sections were stained with haematoxylin and eosin as a routine method and occasionally by the periodic acid Schiff (P.A.S.) method. Frozen sections of liver, kidney and heart were stained for lipids with Sudan IV. The histological and staining procedures followed were according to the Armed Forces Institute of Pathology " Manual of Histological and Special Staining Technics " (1960).

### RESULTS

As the results of acute toxicity in rats, cattle and goats of this experiment have been reported in another paper, the description of the pathological findings will be limited to the neoplastic changes and lesions preceding them. The numbers and types of tumour are shown in the tables.

TABLE I.—*Numbers of Rats and Mortality on Various Diets*

| Percentage of starchy kernel in diet | Initial number of rats at weaning | Number of rats dead at 6 months |
|---|---|---|
| 20 | 40 | 40 |
| 10 | 100 | 73 |
| 5 | 100 | 56 |
| 0 | 50 | 10 killed |

TABLE II.—*Types of Tumour in rats Fed* 10 *per cent Starchy Kernel for* 6 *to* 10 *Months*

| Interval in months | Site of tumour and number of rats involved | Type of tumour |
|---|---|---|
| 6 | (2) Kidney | 2 Adenoma |
| 7 | (3) Kidney | 3 Fibrosarcoma |
| | (7) Liver | 6 Hepatocellular carcinoma |
| | | 5 Cystadenoma |
| | | 2 Fibrosarcoma |
| 8 | (8) Kidney | 7 Fibrosarcoma |
| | | 4 Adenoma |
| | (9) Liver | 6 Hepatocellular carcinoma |
| | | 8 Cystadenoma |
| | | 3 Bile duct adenoma |
| | | 3 Fibrosarcoma |
| | (4) Lung | 4 Adenoma |
| 9+10 | (3) Kidney | 3 Fibrosarcoma |
| | (9) Liver | 7 Hepatocellular carcinoma |
| | | 5 Cystadenoma |
| | | 3 Bile duct adenoma |
| | (3) Lung | 3 Adenoma |

TABLE III.—*Type of Tumours in Rats Fed* 5 *per cent Starchy Flour for* 6 *to* 10 *Months*

| Interval in months | Site of tumour and number of rats involved | Type of tumour |
|---|---|---|
| 6–7 | (7) Kidney | 7 Adenoma |
| | | 1 Fibrosarcoma |
| | (10) Liver | 5 Hepatocellular carcinoma |
| | | 8 Cystadenoma |
| | | 2 Bile duct adenoma |
| 8 | (3) Kidney | 2 Fibrosarcoma |
| | | 1 Adenoma |
| | (10) Liver | 4 Hepatocellular carcinoma |
| | | 2 Fibrosarcoma |
| | | 4 Cystadenoma |
| | | 2 Bile duct adenoma |
| | (3) Lung | 3 Adenoma |
| 9–10 | (7) Kidney | 3 Adenoma |
| | | 4 Fibrosarcoma |
| | (10) Liver | 2 Hepatocellular carcinoma |
| | | 10 Cystadenoma |
| | | 2 Bile duct adenoma |
| | | 1 Fibrosarcoma |

Note: In some rats one liver had three different types of tumour and occasionally one kidney had an adenoma and a fibrosarcoma simultaneously. No tumour was found in the control group.

*Lesions*

There was a high mortality among rats fed 20, 10 and 5 per cent starchy kernel flour. Several rats in the 10 and 5 per cent starchy kernel experimental groups survived beyond the fifth month, and from these the chronic effects of the starchy kernel flour were studied and the lesions which preceded neoplasms were noted.

*Liver.*—The first definite macroscopic changes were seen in a rat dying after 13 weeks on a diet containing 10 per cent starchy kernel flour. The liver had a granular surface, and in addition to the granularity there were several greyish-whitish nodules. All the rats, which survived after 4 months on the experimental diet, without exception had these greyish-whitish nodules scattered over the surface of the liver (Fig. 1, arrow). The number of the nodules varied according to the concentration of the starchy kernel flour in the experimental diet. In addition to the small solid nodules in animals on experimental diets containing 10 and 5 per cent starchy kernel flour, there were translucent cysts on the surface of the liver. The cysts varied in number in different parts of the liver and there were more in rats feeding on the diet containing 10 per cent starchy kernel flour. A tumour of the liver was seen first in a rat fed on the diet containing 10 per cent starchy kernel flour for 7 months, but thereafter the incidence of liver tumours increased.

Grossly, the tumours were whitish-grey in colour with a firm or soft consistency. These tumours were usually multiple, involving one or two of the lobes of the liver. More tumour mass was usually seen in the middle lobe of the liver than in the other lobes. Direct extension to the mesentery and peritoneum was observed (Fig. 2) and metastasis to the lungs was noted in a few cases. The largest of these tumours was oval in shape with a diameter of 5·25 cm. and weighed 56 g.

Histologically, nodular hyperplasia was noted in rats which died after 3 months on the experimental diet. The hyperplastic nodules were composed of closely packed parenchymal cells, which lacked the normal trabecular arrangement and with no sinusoids. The cells forming these nodules were large with enlarged nucleoli. They had acidophilic cytoplasm. The cytoplasm was vacuolated and the vacuoles normally contained no fat. In addition, within the vacuolated cytoplasm in these nodules were many hyaline droplets. The nodules were compressing the surrounding structures and had no capsule around them, but their acidophilic cytoplasm contrasted sharply with the smaller slightly basophilic hepatic cells. Associated with nodular hyperplasia in the early stages was marked bile duct proliferation.

*Tumours of liver*

*Cystadenomas.*—These appeared grossly as translucent cyst-like growths on the liver. Histologically, these tumours consisted of relatively large cavernous spaces lined with flattened endothelial-like cells (Fig. 5). In other places the spaces were lined by low cuboidal epithelium. Between these cavernous spaces there was a stroma made up of slender collagen fibres, and occasionally, normal columns of hepatic cells at different stages of degeneration could be seen in the stroma. These cystic lesions resembled haemangiomas but, instead of blood, they contained serous fluid.

*Bile duct adenomas.*—These tumours grossly were small whitish nodules. Histologically, they were composed of granular structures with their lumens filled with mucous exudate (Fig. 4). The glands were lined with cuboidal epithelium and were separated by wide bands of connective tissue. These tumours in most cases were adjacent to a hepatocellular carcinoma.

*Hepatocellular carcinomas.*—The histological pattern of these tumours varied. In some areas tumours were composed of large cells forming cell sheets. The tumour cells had enlarged nuclei. In other areas the tumours were formed by small cells resembling the normal liver cells but with an abnormal orientation into broad trabeculae (Fig. 3). In some areas there were large sinuses in the centre of the trabeculae. In some places the tumour cells were vacuolated and fat droplets were demonstrated in these vacuolated cells.

*Fibrosarcomas.*—These tumours had a cellular component which showed great pleomorphism (Fig. 6). The cells had scanty cytoplasm and round hyperchromatic nuclei with prominent nucleoli. These cells were closely packed. In some areas cells were spindle-shaped with occasional mitotic figures. In these tumours there were large vascular spaces which resembled dilated sinusoids.

### *Tumours of kidney*

The first gross lesion of the kidney was noted in a rat feeding on a diet containing 10 per cent starchy kernel flour for 30 weeks. The lesion was a small solid whitish-grey nodule in the renal cortex. Rats examined post mortem after the eighth month on the experimental diet had tumours in one or both kidneys.

---

### EXPLANATION OF PLATES

FIG. 1.—Kidneys and liver of a rat fed 10 per cent *Encephalartos hildebrandtii* starchy kernel flour in its diet for 6 months. Normal kidney top right, kidney with renal adenoma top left. The liver in the lower part of the illustration shows greyish hyperplastic nodules, one of which is arrowed.

FIG. 2.—Liver of a rat fed 10 per cent *Encephalartos hildebrandtii* starchy kernel flour in its diet for 8 months. N—Neoplastic masses on the liver. NP—Neoplastic mass on the peritoneum. S—Spleen.

FIG. 3.—A section of hepatocellular carcinoma on the liver of a rat fed 10 per cent *Encephalartos hildebrandtii* starchy kernel flour for 8 months. The tumour mass is formed by cells resembling normal hepatic cells but with cell sheets instead of the column formation of normal liver. H. and E. ×254.

FIG. 4.—Bile duct adenoma adjacent to apparently normal hepatic parenchyma from the liver of a rat fed 5 per cent *Encephalartos hildebrandtii* starchy kernel flour in its diet for 9 months. The adenoma shows papillary formation. H. and E. ×88.

FIG. 5.—A section of cystadenoma in the liver of a rat fed 10 per cent *Encephalartos hildebrandtii* starchy kernel flour in its diet for 7 months. H. and E. ×88.

FIG. 6.—A section of fibrosarcoma of the liver of a rat fed 10 per cent *Encephalartos hildebrandtii* starchy kernel flour in its diet for 9 months. Surviving hepatic cells in lower part of field. H. and E. ×88.

FIG. 7.—A lung of a rat fed 10 per cent *Encephalartos hildebrandtii* starchy kernel flour in its diet for 8 months. N—Neoplastic masses in the lungs.

FIG. 8.—A section of a neoplastic mass in Fig. 7 showing lung adenoma. H. and E. ×125.

FIG. 9.—A section of renal solid adenoma in the kidney of a rat fed 10 per cent *Encephalartos hildebrandtii* starchy kernel flour for 9 months. The adenoma had many narrow fibrous strands dividing it into lobules. H. and E. ×88.

FIG. 10.—A section of cystic adenoma in the kidney of the same rat as in Fig. 9. H. and E. ×88.

FIG. 11.—A section of kidney from a rat fed 5 per cent *Encephalartos hildebrandtii* starchy kernel for 9 months. Normal kidney medulla above, with fibrosarcoma which has replaced normal kidney tissue below. H. and E. ×35.

FIG. 12.—A higher power of Fig. 11 at B showing fibrosarcoma cells with occasional mitotic figures. H. and E. ×88.

FIG. 13.—A section of fibrosarcoma of the kidney in the same rat as in Fig. 11 which had metastesized to the liver. The fibrosarcoma cells in the upper part of the field are infiltrating the hepatic cells below. H. and E. ×88.

1

2

Mugera and Nderito.

3 4 5 6

Mugera and Nderito.

7

8

9

10

11

12

13

Mugera and Nderito.

Some tumours were only visible when the kidneys were sectioned, and then appeared as oval whitish-grey areas in the renal cortex. Some of the tumours were very large and occupied extensive parts of the kidneys, leaving only small areas of the renal tissue and occupying most of the abdominal cavity. These tumours were whitish-grey in colour and on cross section they displayed areas of haemorrhage and necrosis. Metastases were seen in the lungs, liver and peritoneal cavity in 6 cases of renal tumour.

*Adenomas.*—Adenomas in the kidney were of 2 types, solid and cystic papillary adenomas. These tumours were sharply demarcated from the renal parenchyma by their basophilic cells, but they had no capsule. The adjacent tubules appeared compressed and narrow. The neoplastic structure was composed of very closely packed cells with round or oval vesicular nuclei. The cytoplasm of these cells was more deeply basophilic than that of the adjacent tubular cells. There were fine intersecting fibrous strands with capillaries, giving a clear lobular structure to the mass (Fig. 9). In some cases the adenomas had cysts of different sizes (Fig. 10). The cysts were in the centre of a solid mass formed by several cells thick. The cells were similar to those composing the solid adenomas. In a few cases there was papillary projection into the lumen of the cyst.

*Fibrosarcomas.*—These were usually large tumours, with metastasis to the lungs, liver and lymph nodes. They were composed of very anaplastic and pleomorphic cells of fusiform or polyhedral shape. In some areas the tumours showed many whorls and interwoven bundles of immature fibroblasts. The tumour cell nuclei were round and hyperchromatic. Mitotic figures were very common (Fig. 12). Secondary infection and necrosis was very common in these tumours. Among these anaplastic cells there were dilated cavernous spaces lined with a single layer of endothelial cells. These spaces were filled with eosinophilic protein-like material with fibrin strands. The neoplastic cells were often seen infiltrating the normal renal tissue.

### *Tumours of lungs*

Pulmonary tumours were seen in rats after 8 months on the experimental diet. The pulmonary tumours consisted of shiny greyish-white nodules protruding from the surface of the lungs. Their number varied in each case from 3 to 10 nodules. The pulmonary parenchyma around the nodules was congested.

*Adenomas.*—The lung adenomas presented a uniform picture of closely packed columns of cuboidal epithelium (Fig. 8). The cellular element was attached by a basement membrane to the supporting connective tissue stroma. The tumour cells were arranged in an acinar pattern and in some areas they showed papillary formation. No mitotic figures were seen in these tumours and no metastases.

### DISCUSSION

These observations indicate that non-detoxicated starchy kernel flour prepared from *Encephalartos hildebrandtii* contains some toxic and carcinogenic factors which produce tumours of the liver, kidney and lungs. The toxic and carcinogenic factors in this plant have not been identified but could be the same as in *Cycas circinalis*, another cycad group. Matsumoto and Strong (1963) isolated a carcinogenic factor in *Cycas circinalis* and identified it as methylazoxymethoxyglucoside. Laqueur (1964) demonstrated that methylazoxymethanol-glucoside is capable of inducing tumours of the liver, kidney, lungs and intestines.

The tumours in the liver and kidney were similar to those seen in rat fed *Cycas circinalis* flour in the diet for several months (Mugera, Whitehair and Mickelson, 1964; Mugera, 1965; Laqueur *et al.*, 1963). Laqueur *et al.* (1963) reported adenomas of the lungs and intestines in rats fed flour of *Cycas circinalis* in their diet but Mugera *et al.* (1964) did not see any tumour of the lung in rats fed on a similar diet. The adenoma of the lungs seen in these experiments were similar to those reported by Laqueur *et al.* (1963).

The finding of a naturally occurring carcinogen in a plant commonly used by man in East Africa raises a theoretical consideration of the possible association of this plant and cancer in man and suggests a need for carefully conducted epidemiological studies in the population using *Encephalartos hildebrandtii* starch.

Available information suggests a high incidence of liver carcinoma in all areas where cycads are indigenous and are often used. Higginson (1963) summarized epidemiological data on primary liver carcinoma. This summary indicates a high incidence of primary liver cancer in Africa, south of the Sahara, and in South East Asia. The fact that in these same areas cycads are indigenous suggests a possible connection between the two.

### SUMMARY

Neoplasms developed in rats after chronic ingestion of the starchy kernel of the *Encephalartos hildebrandtii* meal as a part of their diet. The tumours developed in the liver, kidneys and lungs. The tumours in the liver were hepatocellular carcinomas, fibrosarcomas, cystadenomas and bile duct adenomas, occasionally occurring simultaneously in the same liver. The tumours in the kidney were mainly fibrosarcomas, with a few adenomas. The adenomas were either cystic or non-cystic. The lung tumours were adenomas.

This work was supported by the East African Medical Research Council.

### REFERENCES

HIGGINSON, J.—(1963) *Cancer Res.*, **23**, 1624.

LAQUEUR, G. L.—(1964) *Fedn Proc. Fedn Am. Socs exp. Biol.*, **23**, 1386.

LAQUEUR, G. L., MICKELSEN, O., WHITING, M. G. AND KURLAND, L. T.—(1963) *J. natn. Cancer Inst.*, **31**, 919.

MATSUMOTO, H. AND STRONG, F. M.—(1963) *Archs Biochem. Biophys.*, **101**, 299.

MUGERA, G. M.—(1965) ' Cycad toxicosis and related carcinogenesis in animals ', Ph.D. Thesis (Michigan State University).

MUGERA, G. M. AND NDERITO, P.—(1967) *E. Afr. med. J.* (in press).

MUGERA, G. M., WHITEHAIR, C. K. AND MICKELSON, O.—(1964) *Fedn Proc. Fedn Am. Socs exp. Biol.*, **23**, 106.